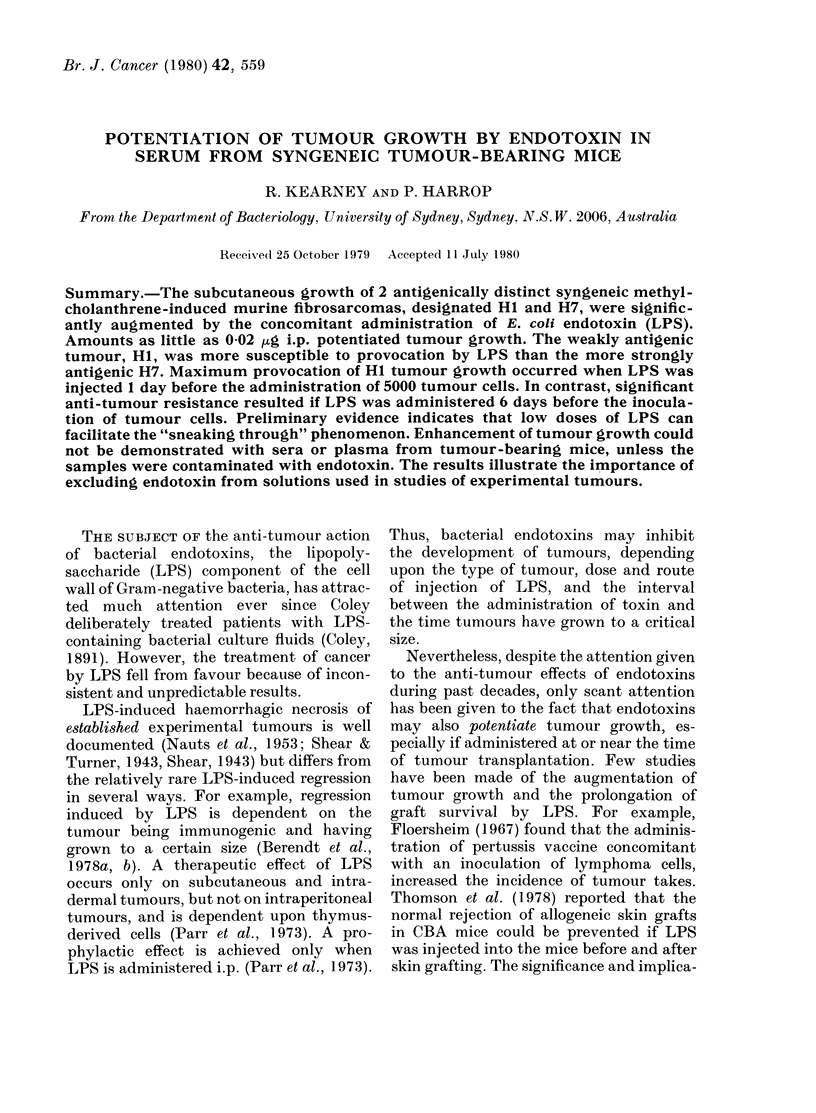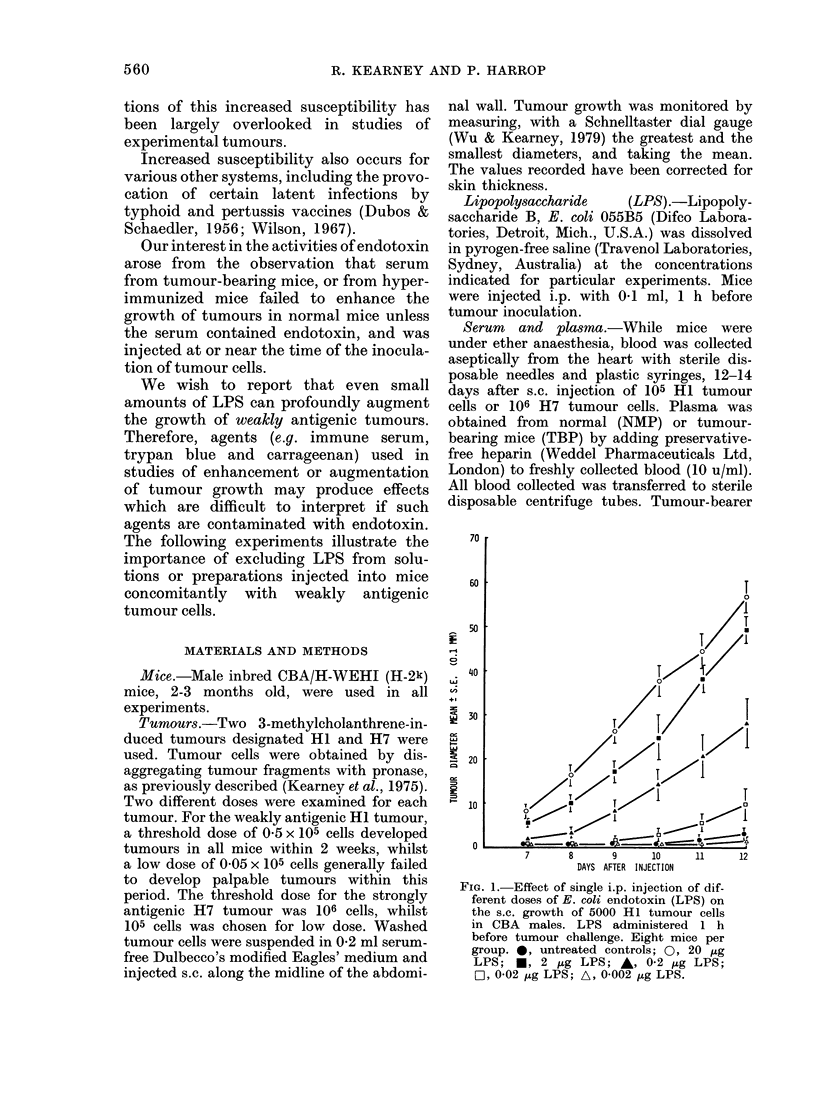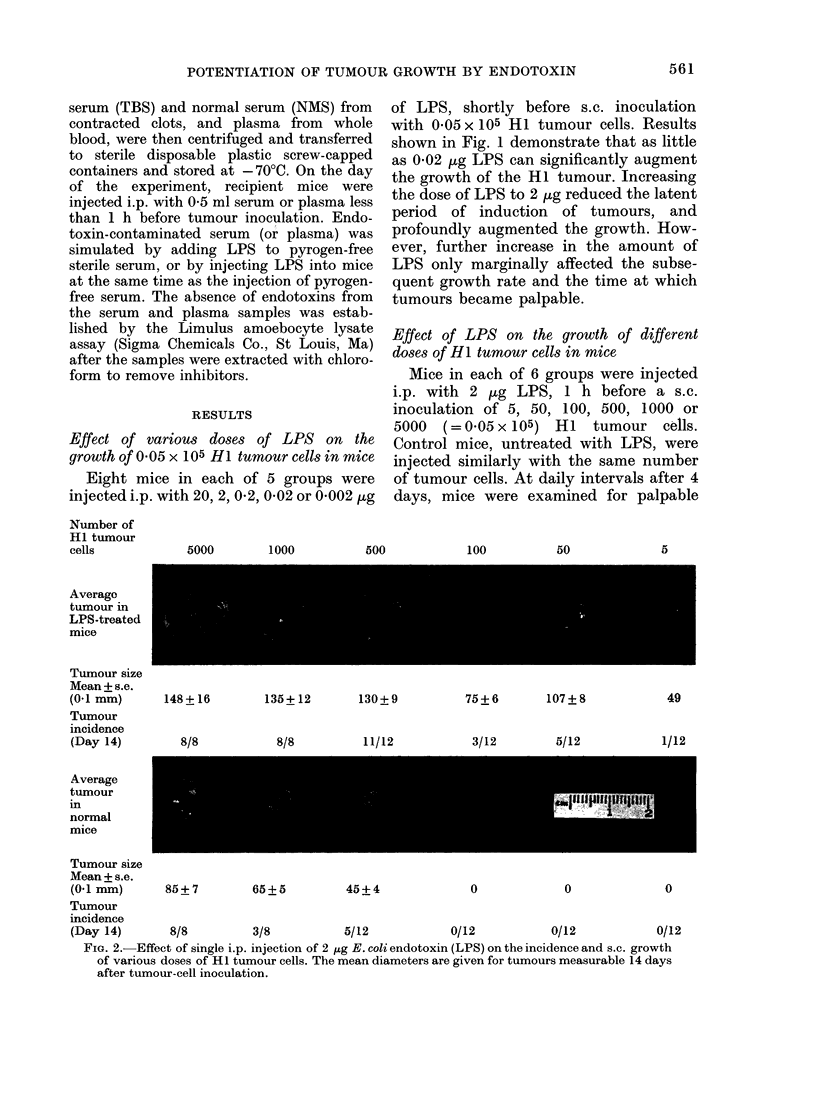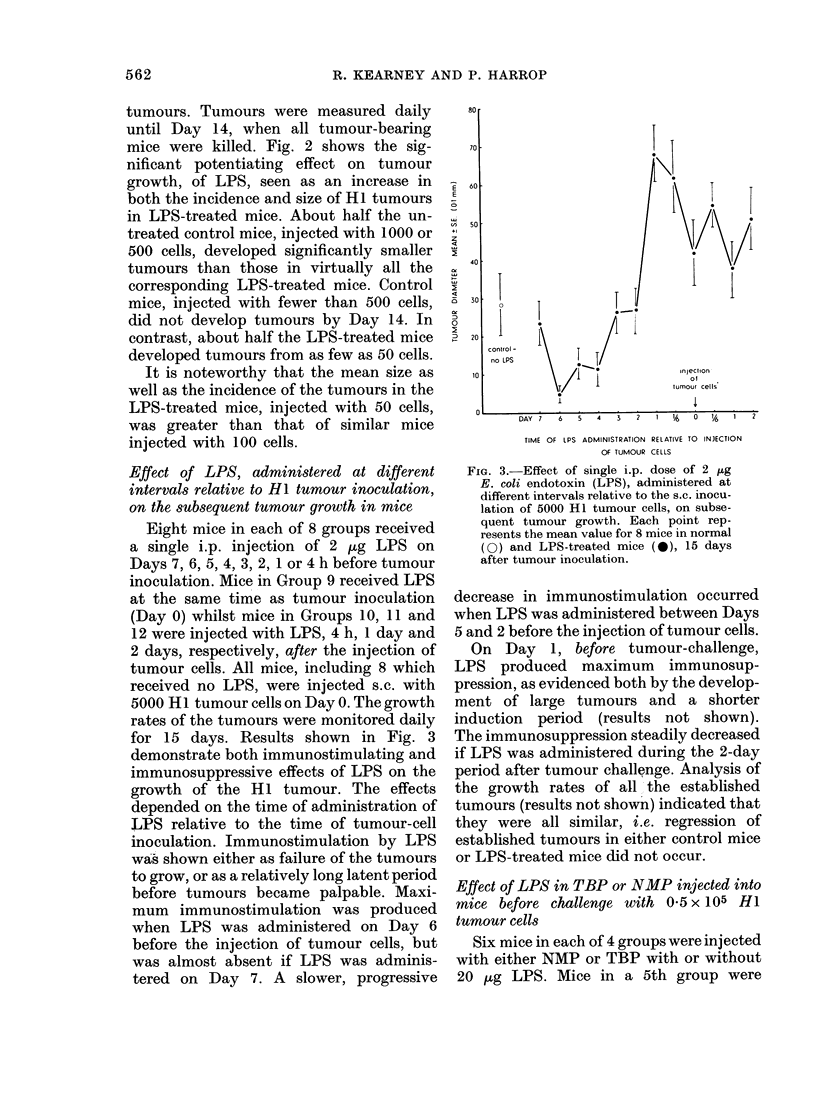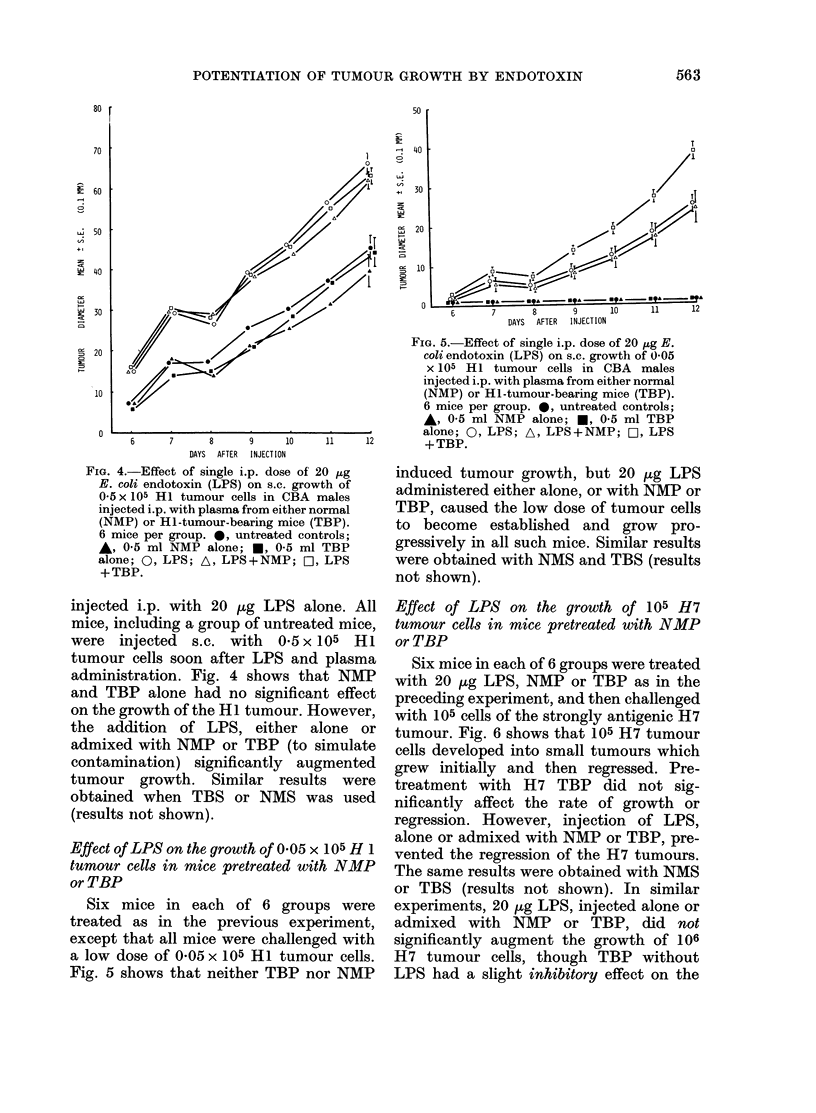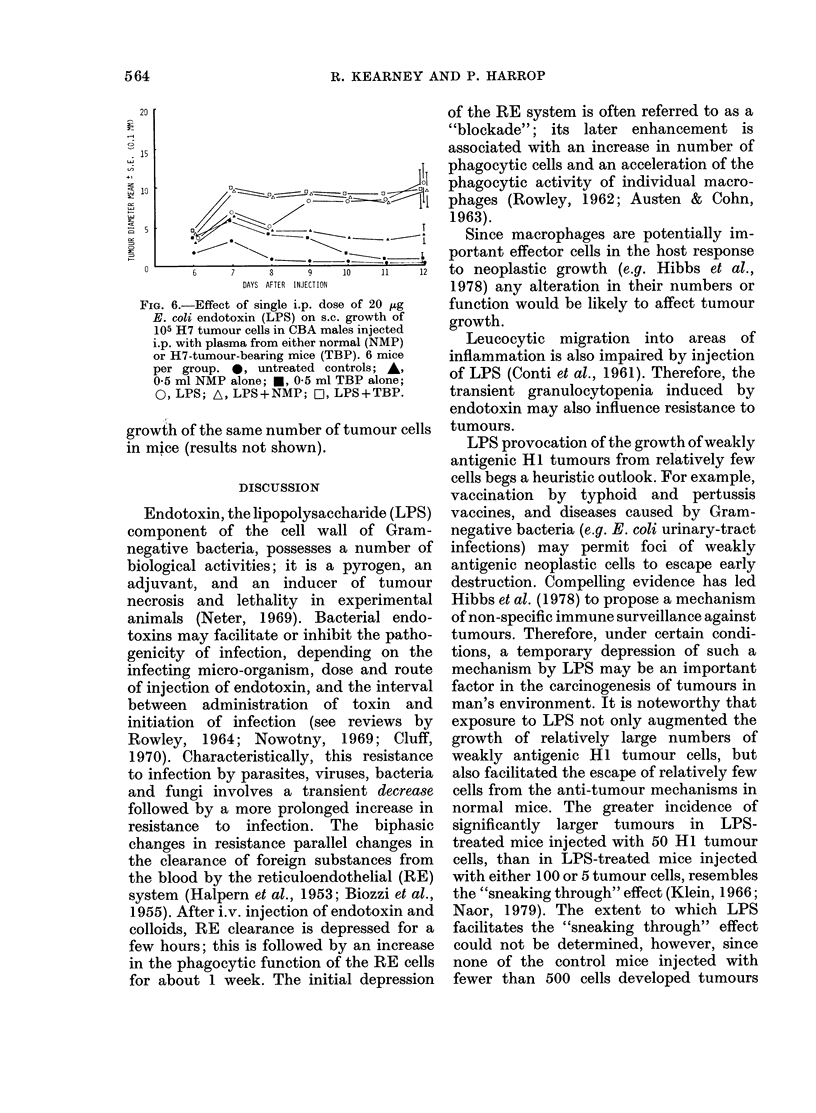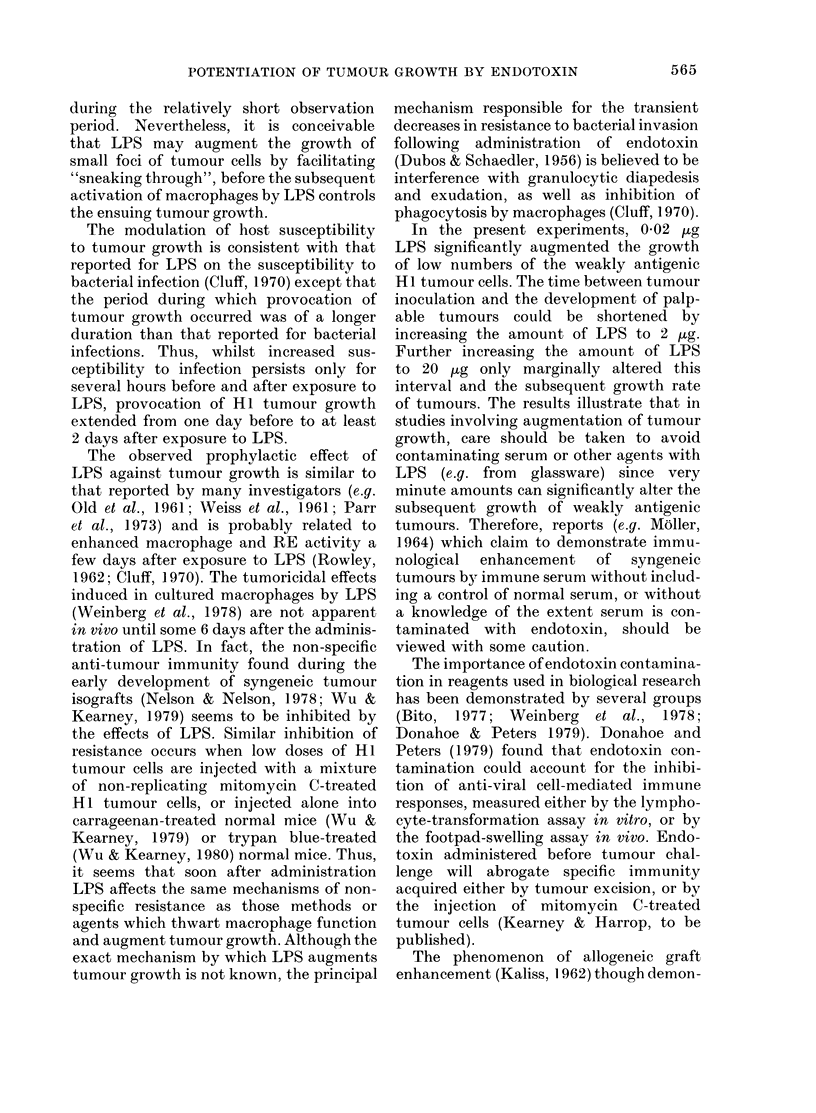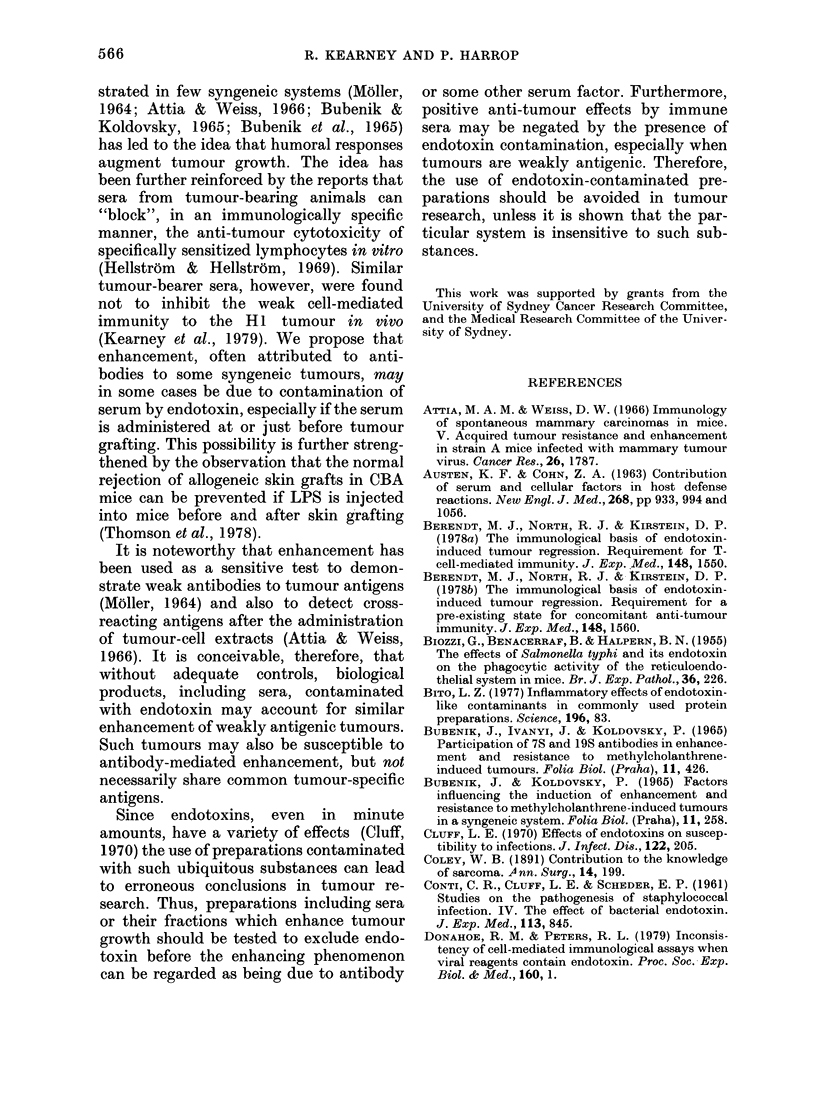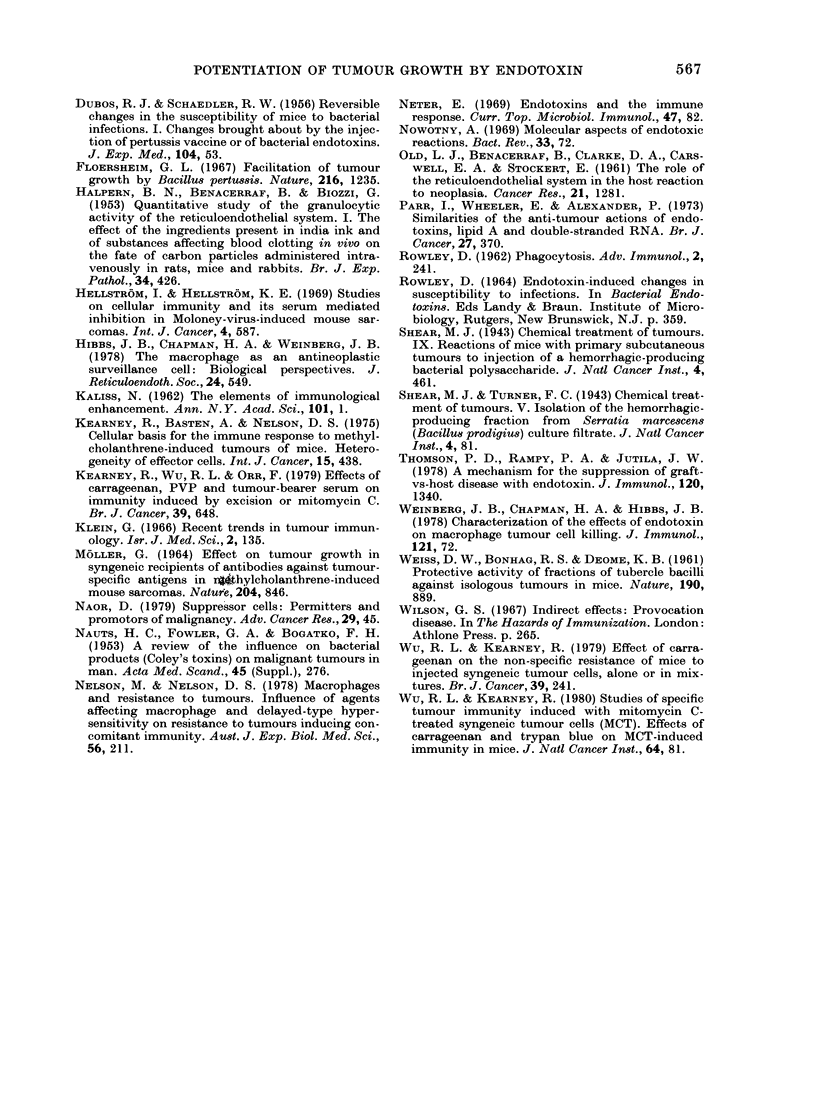# Potentiation of tumour growth by endotoxin in serum from syngeneic tumour-bearing mice.

**DOI:** 10.1038/bjc.1980.280

**Published:** 1980-10

**Authors:** R. Kearney, P. Harrop

## Abstract

**Images:**


*Br. J. Cancer* (1980) **42**, 559

### POTENTIATION OF TUMOUR GROWTH BY ENDOTOXIN IN SERUM FROM SYNGENEIC TUMOUR-BEARING MICE

R. KEARNEY AND P. HARROP

*From the Department of Bacteriology, University of Sydney, Sydney, N.S.W. 2006, Australia*

Received 25 October 1979 Accepted 11 July 1980

**Summary.—The subcutaneous growth of 2 antigenically distinct syngeneic methylcholanthrene-induced murine fibrosarcomas, designated H1 and H7, were significantly augmented by the concomitant administration of *E. coli* endotoxin (LPS). Amounts as little as 0·02 μg i.p. potentiated tumour growth. The weakly antigenic tumour, H1, was more susceptible to provocation by LPS than the more strongly antigenic H7. Maximum provocation of H1 tumour growth occurred when LPS was injected 1 day before the administration of 5000 tumour cells. In contrast, significant anti-tumour resistance resulted if LPS was administered 6 days before the inoculation of tumour cells. Preliminary evidence indicates that low doses of LPS can facilitate the "sneaking through" phenomenon. Enhancement of tumour growth could not be demonstrated with sera or plasma from tumour-bearing mice, unless the samples were contaminated with endotoxin. The results illustrate the importance of excluding endotoxin from solutions used in studies of experimental tumours.**

THE SUBJECT OF the anti-tumour action of bacterial endotoxins, the lipopolysaccharide (LPS) component of the cell wall of Gram-negative bacteria, has attracted much attention ever since Coley deliberately treated patients with LPS-containing bacterial culture fluids (Coley, 1891). However, the treatment of cancer by LPS fell from favour because of inconsistent and unpredictable results.

LPS-induced haemorrhagic necrosis of *established* experimental tumours is well documented (Nauts *et al.*, 1953; Shear & Turner, 1943, Shear, 1943) but differs from the relatively rare LPS-induced regression in several ways. For example, regression induced by LPS is dependent on the tumour being immunogenic and having grown to a certain size (Berendt *et al.*, 1978*a*, *b*). A therapeutic effect of LPS occurs only on subcutaneous and intradermal tumours, but not on intraperitoneal tumours, and is dependent upon thymus-derived cells (Parr *et al.*, 1973). A prophylactic effect is achieved only when LPS is administered i.p. (Parr *et al.*, 1973). Thus, bacterial endotoxins may inhibit the development of tumours, depending upon the type of tumour, dose and route of injection of LPS, and the interval between the administration of toxin and the time tumours have grown to a critical size.

Nevertheless, despite the attention given to the anti-tumour effects of endotoxins during past decades, only scant attention has been given to the fact that endotoxins may also *potentiate* tumour growth, especially if administered at or near the time of tumour transplantation. Few studies have been made of the augmentation of tumour growth and the prolongation of graft survival by LPS. For example, Floersheim (1967) found that the administration of pertussis vaccine concomitant with an inoculation of lymphoma cells, increased the incidence of tumour takes. Thomson *et al.* (1978) reported that the normal rejection of allogeneic skin grafts in CBA mice could be prevented if LPS was injected into the mice before and after skin grafting. The significance and implications of this increased susceptibility has been largely overlooked in studies of experimental tumours.

Increased susceptibility also occurs for various other systems, including the provocation of certain latent infections by typhoid and pertussis vaccines (Dubos & Schaedler, 1956; Wilson, 1967).

Our interest in the activities of endotoxin arose from the observation that serum from tumour-bearing mice, or from hyperimmunized mice failed to enhance the growth of tumours in normal mice unless the serum contained endotoxin, and was injected at or near the time of the inoculation of tumour cells.

We wish to report that even small amounts of LPS can profoundly augment the growth of *weakly* antigenic tumours. Therefore, agents (*e.g.* immune serum, trypan blue and carrageenan) used in studies of enhancement or augmentation of tumour growth may produce effects which are difficult to interpret if such agents are contaminated with endotoxin. The following experiments illustrate the importance of excluding LPS from solutions or preparations injected into mice concomitantly with weakly antigenic tumour cells.

### MATERIALS AND METHODS

*Mice.*—Male inbred CBA/H-WEHI (H-$2^k$) mice, 2-3 months old, were used in all experiments.

*Tumours.*—Two 3-methylcholanthrene-induced tumours designated H1 and H7 were used. Tumour cells were obtained by disaggregating tumour fragments with pronase, as previously described (Kearney *et al.*, 1975). Two different doses were examined for each tumour. For the weakly antigenic H1 tumour, a threshold dose of $0{\cdot}5 \times 10^5$ cells developed tumours in all mice within 2 weeks, whilst a low dose of $0{\cdot}05 \times 10^5$ cells generally failed to develop palpable tumours within this period. The threshold dose for the strongly antigenic H7 tumour was $10^6$ cells, whilst $10^5$ cells was chosen for low dose. Washed tumour cells were suspended in 0·2 ml serum-free Dulbecco's modified Eagles' medium and injected s.c. along the midline of the abdominal wall. Tumour growth was monitored by measuring, with a Schnelltaster dial gauge (Wu & Kearney, 1979) the greatest and the smallest diameters, and taking the mean. The values recorded have been corrected for skin thickness.

*Lipopolysaccharide* (*LPS*).—Lipopolysaccharide B, *E. coli* 055B5 (Difco Laboratories, Detroit, Mich., U.S.A.) was dissolved in pyrogen-free saline (Travenol Laboratories, Sydney, Australia) at the concentrations indicated for particular experiments. Mice were injected i.p. with 0·1 ml, 1 h before tumour inoculation.

*Serum and plasma.*—While mice were under ether anaesthesia, blood was collected aseptically from the heart with sterile disposable needles and plastic syringes, 12–14 days after s.c. injection of $10^5$ H1 tumour cells or $10^6$ H7 tumour cells. Plasma was obtained from normal (NMP) or tumour-bearing mice (TBP) by adding preservative-free heparin (Weddel Pharmaceuticals Ltd, London) to freshly collected blood (10 u/ml). All blood collected was transferred to sterile disposable centrifuge tubes. Tumour-bearer

FIG. 1.—Effect of single i.p. injection of different doses of *E. coli* endotoxin (LPS) on the s.c. growth of 5000 H1 tumour cells in CBA males. LPS administered 1 h before tumour challenge. Eight mice per group. ●, untreated controls; ○, 20 μg LPS; ■, 2 μg LPS; ▲, 0·2 μg LPS; □, 0·02 μg LPS; △, 0·002 μg LPS.

serum (TBS) and normal serum (NMS) from contracted clots, and plasma from whole blood, were then centrifuged and transferred to sterile disposable plastic screw-capped containers and stored at −70°C. On the day of the experiment, recipient mice were injected i.p. with 0·5 ml serum or plasma less than 1 h before tumour inoculation. Endotoxin-contaminated serum (or plasma) was simulated by adding LPS to pyrogen-free sterile serum, or by injecting LPS into mice at the same time as the injection of pyrogen-free serum. The absence of endotoxins from the serum and plasma samples was established by the Limulus amoebocyte lysate assay (Sigma Chemicals Co., St Louis, Ma) after the samples were extracted with chloroform to remove inhibitors.

### RESULTS

### *Effect of various doses of LPS on the growth of* $0{\cdot}05 \times 10^5$ *H1 tumour cells in mice*

Eight mice in each of 5 groups were injected i.p. with 20, 2, 0·2, 0·02 or 0·002 μg of LPS, shortly before s.c. inoculation with $0{\cdot}05 \times 10^5$ H1 tumour cells. Results shown in Fig. 1 demonstrate that as little as 0·02 μg LPS can significantly augment the growth of the H1 tumour. Increasing the dose of LPS to 2 μg reduced the latent period of induction of tumours, and profoundly augmented the growth. However, further increase in the amount of LPS only marginally affected the subsequent growth rate and the time at which tumours became palpable.

### *Effect of LPS on the growth of different doses of H1 tumour cells in mice*

Mice in each of 6 groups were injected i.p. with 2 μg LPS, 1 h before a s.c. inoculation of 5, 50, 100, 500, 1000 or 5000 ($=0{\cdot}05 \times 10^5$) H1 tumour cells. Control mice, untreated with LPS, were injected similarly with the same number of tumour cells. At daily intervals after 4 days, mice were examined for palpable

FIG. 2.—Effect of single i.p. injection of 2 μg *E. coli* endotoxin (LPS) on the incidence and s.c. growth of various doses of H1 tumour cells. The mean diameters are given for tumours measurable 14 days after tumour-cell inoculation.

tumours. Tumours were measured daily until Day 14, when all tumour-bearing mice were killed. Fig. 2 shows the significant potentiating effect on tumour growth, of LPS, seen as an increase in both the incidence and size of H1 tumours in LPS-treated mice. About half the untreated control mice, injected with 1000 or 500 cells, developed significantly smaller tumours than those in virtually all the corresponding LPS-treated mice. Control mice, injected with fewer than 500 cells, did not develop tumours by Day 14. In contrast, about half the LPS-treated mice developed tumours from as few as 50 cells.

It is noteworthy that the mean size as well as the incidence of the tumours in the LPS-treated mice, injected with 50 cells, was greater than that of similar mice injected with 100 cells.

*Effect of LPS, administered at different intervals relative to H1 tumour inoculation, on the subsequent tumour growth in mice*

Eight mice in each of 8 groups received a single i.p. injection of 2 μg LPS on Days 7, 6, 5, 4, 3, 2, 1 or 4 h before tumour inoculation. Mice in Group 9 received LPS at the same time as tumour inoculation (Day 0) whilst mice in Groups 10, 11 and 12 were injected with LPS, 4 h, 1 day and 2 days, respectively, *after* the injection of tumour cells. All mice, including 8 which received no LPS, were injected s.c. with 5000 H1 tumour cells on Day 0. The growth rates of the tumours were monitored daily for 15 days. Results shown in Fig. 3 demonstrate both immunostimulating and immunosuppressive effects of LPS on the growth of the H1 tumour. The effects depended on the time of administration of LPS relative to the time of tumour-cell inoculation. Immunostimulation by LPS was shown either as failure of the tumours to grow, or as a relatively long latent period before tumours became palpable. Maximum immunostimulation was produced when LPS was administered on Day 6 before the injection of tumour cells, but was almost absent if LPS was administered on Day 7. A slower, progressive

FIG. 3.—Effect of single i.p. dose of 2 μg *E. coli* endotoxin (LPS), administered at different intervals relative to the s.c. inoculation of 5000 H1 tumour cells, on subsequent tumour growth. Each point represents the mean value for 8 mice in normal (○) and LPS-treated mice (●), 15 days after tumour inoculation.

decrease in immunostimulation occurred when LPS was administered between Days 5 and 2 before the injection of tumour cells.

On Day 1, *before* tumour-challenge, LPS produced maximum immunosuppression, as evidenced both by the development of large tumours and a shorter induction period (results not shown). The immunosuppression steadily decreased if LPS was administered during the 2-day period after tumour challenge. Analysis of the growth rates of all the established tumours (results not shown) indicated that they were all similar, *i.e.* regression of established tumours in either control mice or LPS-treated mice did not occur.

*Effect of LPS in TBP or NMP injected into mice before challenge with* $0{\cdot}5 \times 10^5$ *H1 tumour cells*

Six mice in each of 4 groups were injected with either NMP or TBP with or without 20 μg LPS. Mice in a 5th group were

FIG. 4.—Effect of single i.p. dose of 20 μg *E. coli* endotoxin (LPS) on s.c. growth of 0·5 × $10^5$ H1 tumour cells in CBA males injected i.p. with plasma from either normal (NMP) or H1-tumour-bearing mice (TBP). 6 mice per group. ●, untreated controls; ▲, 0·5 ml NMP alone; ■, 0·5 ml TBP alone; ○, LPS; △, LPS+NMP; □, LPS +TBP.

FIG. 5.—Effect of single i.p. dose of 20 μg *E. coli* endotoxin (LPS) on s.c. growth of 0·05 × $10^5$ H1 tumour cells in CBA males injected i.p. with plasma from either normal (NMP) or H1-tumour-bearing mice (TBP). 6 mice per group. ●, untreated controls; ▲, 0·5 ml NMP alone; ■, 0·5 ml TBP alone; ○, LPS; △, LPS+NMP; □, LPS +TBP.

injected i.p. with 20 μg LPS alone. All mice, including a group of untreated mice, were injected s.c. with 0·5 × $10^5$ H1 tumour cells soon after LPS and plasma administration. Fig. 4 shows that NMP and TBP alone had no significant effect on the growth of the H1 tumour. However, the addition of LPS, either alone or admixed with NMP or TBP (to simulate contamination) significantly augmented tumour growth. Similar results were obtained when TBS or NMS was used (results not shown).

*Effect of LPS on the growth of* 0·05 × $10^5$ *H* 1 *tumour cells in mice pretreated with NMP or TBP*

Six mice in each of 6 groups were treated as in the previous experiment, except that all mice were challenged with a low dose of 0·05 × $10^5$ H1 tumour cells. Fig. 5 shows that neither TBP nor NMP induced tumour growth, but 20 μg LPS administered either alone, or with NMP or TBP, caused the low dose of tumour cells to become established and grow progressively in all such mice. Similar results were obtained with NMS and TBS (results not shown).

*Effect of LPS on the growth of* $10^5$ *H*7 *tumour cells in mice pretreated with NMP or TBP*

Six mice in each of 6 groups were treated with 20 μg LPS, NMP or TBP as in the preceding experiment, and then challenged with $10^5$ cells of the strongly antigenic H7 tumour. Fig. 6 shows that $10^5$ H7 tumour cells developed into small tumours which grew initially and then regressed. Pretreatment with H7 TBP did not significantly affect the rate of growth or regression. However, injection of LPS, alone or admixed with NMP or TBP, prevented the regression of the H7 tumours. The same results were obtained with NMS or TBS (results not shown). In similar experiments, 20 μg LPS, injected alone or admixed with NMP or TBP, did *not* significantly augment the growth of $10^6$ H7 tumour cells, though TBP without LPS had a slight *inhibitory* effect on the

FIG. 6.—Effect of single i.p. dose of 20 μg *E. coli* endotoxin (LPS) on s.c. growth of $10^5$ H7 tumour cells in CBA males injected i.p. with plasma from either normal (NMP) or H7-tumour-bearing mice (TBP). 6 mice per group. ●, untreated controls; ▲, 0·5 ml NMP alone; ■, 0·5 ml TBP alone; ○, LPS; △, LPS+NMP; □, LPS+TBP.

growth of the same number of tumour cells in mice (results not shown).

DISCUSSION

Endotoxin, the lipopolysaccharide (LPS) component of the cell wall of Gram-negative bacteria, possesses a number of biological activities; it is a pyrogen, an adjuvant, and an inducer of tumour necrosis and lethality in experimental animals (Neter, 1969). Bacterial endotoxins may facilitate or inhibit the pathogenicity of infection, depending on the infecting micro-organism, dose and route of injection of endotoxin, and the interval between administration of toxin and initiation of infection (see reviews by Rowley, 1964; Nowotny, 1969; Cluff, 1970). Characteristically, this resistance to infection by parasites, viruses, bacteria and fungi involves a transient *decrease* followed by a more prolonged increase in resistance to infection. The biphasic changes in resistance parallel changes in the clearance of foreign substances from the blood by the reticuloendothelial (RE) system (Halpern *et al.*, 1953; Biozzi *et al.*, 1955). After i.v. injection of endotoxin and colloids, RE clearance is depressed for a few hours; this is followed by an increase in the phagocytic function of the RE cells for about 1 week. The initial depression of the RE system is often referred to as a "blockade"; its later enhancement is associated with an increase in number of phagocytic cells and an acceleration of the phagocytic activity of individual macrophages (Rowley, 1962; Austen & Cohn, 1963).

Since macrophages are potentially important effector cells in the host response to neoplastic growth (*e.g.* Hibbs *et al.*, 1978) any alteration in their numbers or function would be likely to affect tumour growth.

Leucocytic migration into areas of inflammation is also impaired by injection of LPS (Conti *et al.*, 1961). Therefore, the transient granulocytopenia induced by endotoxin may also influence resistance to tumours.

LPS provocation of the growth of weakly antigenic H1 tumours from relatively few cells begs a heuristic outlook. For example, vaccination by typhoid and pertussis vaccines, and diseases caused by Gram-negative bacteria (*e.g. E. coli* urinary-tract infections) may permit foci of weakly antigenic neoplastic cells to escape early destruction. Compelling evidence has led Hibbs *et al.* (1978) to propose a mechanism of non-specific immune surveillance against tumours. Therefore, under certain conditions, a temporary depression of such a mechanism by LPS may be an important factor in the carcinogenesis of tumours in man's environment. It is noteworthy that exposure to LPS not only augmented the growth of relatively large numbers of weakly antigenic H1 tumour cells, but also facilitated the escape of relatively few cells from the anti-tumour mechanisms in normal mice. The greater incidence of significantly larger tumours in LPS-treated mice injected with 50 H1 tumour cells, than in LPS-treated mice injected with either 100 or 5 tumour cells, resembles the "sneaking through" effect (Klein, 1966; Naor, 1979). The extent to which LPS facilitates the "sneaking through" effect could not be determined, however, since none of the control mice injected with fewer than 500 cells developed tumours during the relatively short observation period. Nevertheless, it is conceivable that LPS may augment the growth of small foci of tumour cells by facilitating "sneaking through", before the subsequent activation of macrophages by LPS controls the ensuing tumour growth.

The modulation of host susceptibility to tumour growth is consistent with that reported for LPS on the susceptibility to bacterial infection (Cluff, 1970) except that the period during which provocation of tumour growth occurred was of a longer duration than that reported for bacterial infections. Thus, whilst increased susceptibility to infection persists only for several hours before and after exposure to LPS, provocation of H1 tumour growth extended from one day before to at least 2 days after exposure to LPS.

The observed prophylactic effect of LPS against tumour growth is similar to that reported by many investigators (*e.g.* Old *et al.*, 1961; Weiss *et al.*, 1961; Parr *et al.*, 1973) and is probably related to enhanced macrophage and RE activity a few days after exposure to LPS (Rowley, 1962; Cluff, 1970). The tumoricidal effects induced in cultured macrophages by LPS (Weinberg *et al.*, 1978) are not apparent *in vivo* until some 6 days after the administration of LPS. In fact, the non-specific anti-tumour immunity found during the early development of syngeneic tumour isografts (Nelson & Nelson, 1978; Wu & Kearney, 1979) seems to be inhibited by the effects of LPS. Similar inhibition of resistance occurs when low doses of H1 tumour cells are injected with a mixture of non-replicating mitomycin C-treated H1 tumour cells, or injected alone into carrageenan-treated normal mice (Wu & Kearney, 1979) or trypan blue-treated (Wu & Kearney, 1980) normal mice. Thus, it seems that soon after administration LPS affects the same mechanisms of non-specific resistance as those methods or agents which thwart macrophage function and augment tumour growth. Although the exact mechanism by which LPS augments tumour growth is not known, the principal mechanism responsible for the transient decreases in resistance to bacterial invasion following administration of endotoxin (Dubos & Schaedler, 1956) is believed to be interference with granulocytic diapedesis and exudation, as well as inhibition of phagocytosis by macrophages (Cluff, 1970).

In the present experiments, 0·02 $\mu$g LPS significantly augmented the growth of low numbers of the weakly antigenic H1 tumour cells. The time between tumour inoculation and the development of palpable tumours could be shortened by increasing the amount of LPS to 2 $\mu$g. Further increasing the amount of LPS to 20 $\mu$g only marginally altered this interval and the subsequent growth rate of tumours. The results illustrate that in studies involving augmentation of tumour growth, care should be taken to avoid contaminating serum or other agents with LPS (*e.g.* from glassware) since very minute amounts can significantly alter the subsequent growth of weakly antigenic tumours. Therefore, reports (*e.g.* Möller, 1964) which claim to demonstrate immunological enhancement of syngeneic tumours by immune serum without including a control of normal serum, or without a knowledge of the extent serum is contaminated with endotoxin, should be viewed with some caution.

The importance of endotoxin contamination in reagents used in biological research has been demonstrated by several groups (Bito, 1977; Weinberg *et al.*, 1978; Donahoe & Peters 1979). Donahoe and Peters (1979) found that endotoxin contamination could account for the inhibition of anti-viral cell-mediated immune responses, measured either by the lymphocyte-transformation assay *in vitro*, or by the footpad-swelling assay *in vivo*. Endotoxin administered before tumour challenge will abrogate specific immunity acquired either by tumour excision, or by the injection of mitomycin C-treated tumour cells (Kearney & Harrop, to be published).

The phenomenon of allogeneic graft enhancement (Kaliss, 1962) though demonstrated in few syngeneic systems (Möller, 1964; Attia & Weiss, 1966; Bubenik & Koldovsky, 1965; Bubenik *et al.*, 1965) has led to the idea that humoral responses augment tumour growth. The idea has been further reinforced by the reports that sera from tumour-bearing animals can "block", in an immunologically specific manner, the anti-tumour cytotoxicity of specifically sensitized lymphocytes *in vitro* (Hellström & Hellström, 1969). Similar tumour-bearer sera, however, were found not to inhibit the weak cell-mediated immunity to the H1 tumour *in vivo* (Kearney *et al.*, 1979). We propose that enhancement, often attributed to antibodies to some syngeneic tumours, *may* in some cases be due to contamination of serum by endotoxin, especially if the serum is administered at or just before tumour grafting. This possibility is further strengthened by the observation that the normal rejection of allogeneic skin grafts in CBA mice can be prevented if LPS is injected into mice before and after skin grafting (Thomson *et al.*, 1978).

It is noteworthy that enhancement has been used as a sensitive test to demonstrate weak antibodies to tumour antigens (Möller, 1964) and also to detect cross-reacting antigens after the administration of tumour-cell extracts (Attia & Weiss, 1966). It is conceivable, therefore, that without adequate controls, biological products, including sera, contaminated with endotoxin may account for similar enhancement of weakly antigenic tumours. Such tumours may also be susceptible to antibody-mediated enhancement, but *not* necessarily share common tumour-specific antigens.

Since endotoxins, even in minute amounts, have a variety of effects (Cluff, 1970) the use of preparations contaminated with such ubiquitous substances can lead to erroneous conclusions in tumour research. Thus, preparations including sera or their fractions which enhance tumour growth should be tested to exclude endotoxin before the enhancing phenomenon can be regarded as being due to antibody or some other serum factor. Furthermore, positive anti-tumour effects by immune sera may be negated by the presence of endotoxin contamination, especially when tumours are weakly antigenic. Therefore, the use of endotoxin-contaminated preparations should be avoided in tumour research, unless it is shown that the particular system is insensitive to such substances.

This work was supported by grants from the University of Sydney Cancer Research Committee, and the Medical Research Committee of the University of Sydney.

### REFERENCES

ATTIA, M. A. M. & WEISS, D. W. (1966) Immunology of spontaneous mammary carcinomas in mice. V. Acquired tumour resistance and enhancement in strain A mice infected with mammary tumour virus. *Cancer Res.*, **26**, 1787.

AUSTEN, K. F. & COHN, Z. A. (1963) Contribution of serum and cellular factors in host defense reactions. *New Engl. J. Med.*, **268**, pp 933, 994 and 1056.

BERENDT, M. J., NORTH, R. J. & KIRSTEIN, D. P. (1978*a*) The immunological basis of endotoxin-induced tumour regression. Requirement for T-cell-mediated immunity. *J. Exp. Med.*, **148**, 1550.

BERENDT, M. J., NORTH, R. J. & KIRSTEIN, D. P. (1978*b*) The immunological basis of endotoxin-induced tumour regression. Requirement for a pre-existing state for concomitant anti-tumour immunity. *J. Exp. Med.*, **148**, 1560.

BIOZZI, G., BENACERRAF, B. & HALPERN, B. N. (1955) The effects of *Salmonella typhi* and its endotoxin on the phagocytic activity of the reticuloendothelial system in mice. *Br. J. Exp. Pathol.*, **36**, 226.

BITO, L. Z. (1977) Inflammatory effects of endotoxin-like contaminants in commonly used protein preparations. *Science*, **196**, 83.

BUBENIK, J., IVANYI, J. & KOLDOVSKY, P. (1965) Participation of 7S and 19S antibodies in enhancement and resistance to methylcholanthrene-induced tumours. *Folia Biol.* (*Praha*), **11**, 426.

BUBENIK, J. & KOLDOVSKY, P. (1965) Factors influencing the induction of enhancement and resistance to methylcholanthrene-induced tumours in a syngeneic system. *Folia Biol.* (Praha), **11**, 258.

CLUFF, L. E. (1970) Effects of endotoxins on susceptibility to infections. *J. Infect. Dis.*, **122**, 205.

COLEY, W. B. (1891) Contribution to the knowledge of sarcoma. *Ann. Surg.*, **14**, 199.

CONTI, C. R., CLUFF, L. E. & SCHEDER, E. P. (1961) Studies on the pathogenesis of staphylococcal infection. IV. The effect of bacterial endotoxin. *J. Exp. Med.*, **113**, 845.

DONAHOE, R. M. & PETERS, R. L. (1979) Inconsistency of cell-mediated immunological assays when viral reagents contain endotoxin. *Proc. Soc. Exp. Biol. & Med.*, **160**, 1.

DUBOS, R. J. & SCHAEDLER, R. W. (1956) Reversible changes in the susceptibility of mice to bacterial infections. I. Changes brought about by the injection of pertussis vaccine or of bacterial endotoxins. *J. Exp. Med.*, **104**, 53.

FLOERSHEIM, G. L. (1967) Facilitation of tumour growth by *Bacillus pertussis*. *Nature*, **216**, 1235.

HALPERN, B. N., BENACERRAF, B. & BIOZZI, G. (1953) Quantitative study of the granulocytic activity of the reticuloendothelial system. I. The effect of the ingredients present in india ink and of substances affecting blood clotting *in vivo* on the fate of carbon particles administered intravenously in rats, mice and rabbits. *Br. J. Exp. Pathol.*, **34**, 426.

HELLSTRÖM, I. & HELLSTRÖM, K. E. (1969) Studies on cellular immunity and its serum mediated inhibition in Moloney-virus-induced mouse sarcomas. *Int. J. Cancer*, **4**, 587.

HIBBS, J. B., CHAPMAN, H. A. & WEINBERG, J. B. (1978) The macrophage as an antineoplastic surveillance cell: Biological perspectives. *J. Reticuloendoth. Soc.*, **24**, 549.

KALISS, N. (1962) The elements of immunological enhancement. *Ann. N.Y. Acad. Sci.*, **101**, 1.

KEARNEY, R., BASTEN, A. & NELSON, D. S. (1975) Cellular basis for the immune response to methylcholanthrene-induced tumours of mice. Heterogeneity of effector cells. *Int. J. Cancer*, **15**, 438.

KEARNEY, R., WU, R. L. & ORR, F. (1979) Effects of carrageenan, PVP and tumour-bearer serum on immunity induced by excision or mitomycin C. *Br. J. Cancer*, **39**, 648.

KLEIN, G. (1966) Recent trends in tumour immunology. *Isr. J. Med. Sci.*, **2**, 135.

MÖLLER, G. (1964) Effect on tumour growth in syngeneic recipients of antibodies against tumour-specific antigens in methylcholanthrene-induced mouse sarcomas. *Nature*, **204**, 846.

NAOR, D. (1979) Suppressor cells: Permitters and promotors of malignancy. *Adv. Cancer Res.*, **29**, 45.

NAUTS, H. C., FOWLER, G. A. & BOGATKO, F. H. (1953) A review of the influence on bacterial products (Coley's toxins) on malignant tumours in man. *Acta Med. Scand.*, **45** (Suppl.), 276.

NELSON, M. & NELSON, D. S. (1978) Macrophages and resistance to tumours. Influence of agents affecting macrophage and delayed-type hypersensitivity on resistance to tumours inducing concomitant immunity. *Aust. J. Exp. Biol. Med. Sci.*, **56**, 211.

NETER, E. (1969) Endotoxins and the immune response. *Curr. Top. Microbiol. Immunol.*, **47**, 82.

NOWOTNY, A. (1969) Molecular aspects of endotoxic reactions. *Bact. Rev.*, **33**, 72.

OLD, L. J., BENACERRAF, B., CLARKE, D. A., CARSWELL, E. A. & STOCKERT, E. (1961) The role of the reticuloendothelial system in the host reaction to neoplasia. *Cancer Res.*, **21**, 1281.

PARR, I., WHEELER, E. & ALEXANDER, P. (1973) Similarities of the anti-tumour actions of endotoxins, lipid A and double-stranded RNA. *Br. J. Cancer*, **27**, 370.

ROWLEY, D. (1962) Phagocytosis. *Adv. Immunol.*, **2**, 241.

ROWLEY, D. (1964) Endotoxin-induced changes in susceptibility to infections. In *Bacterial Endotoxins*. Eds Landy & Braun. Institute of Microbiology, Rutgers, New Brunswick, N.J. p. 359.

SHEAR, M. J. (1943) Chemical treatment of tumours. IX. Reactions of mice with primary subcutaneous tumours to injection of a hemorrhagic-producing bacterial polysaccharide. *J. Natl Cancer Inst.*, **4**, 461.

SHEAR, M. J. & TURNER, F. C. (1943) Chemical treatment of tumours. V. Isolation of the hemorrhagic-producing fraction from *Serratia marcescens* (*Bacillus prodigius*) culture filtrate. *J. Natl Cancer Inst.*, **4**, 81.

THOMSON, P. D., RAMPY, P. A. & JUTILA, J. W. (1978) A mechanism for the suppression of graft-vs-host disease with endotoxin. *J. Immunol.*, **120**, 1340.

WEINBERG, J. B., CHAPMAN, H. A. & HIBBS, J. B. (1978) Characterization of the effects of endotoxin on macrophage tumour cell killing. *J. Immunol.*, **121**, 72.

WEISS, D. W., BONHAG, R. S. & DEOME, K. B. (1961) Protective activity of fractions of tubercle bacilli against isologous tumours in mice. *Nature*, **190**, 889.

WILSON, G. S. (1967) Indirect effects: Provocation disease. In *The Hazards of Immunization*. London: Athlone Press. p. 265.

WU, R. L. & KEARNEY, R. (1979) Effect of carrageenan on the non-specific resistance of mice to injected syngeneic tumour cells, alone or in mixtures. *Br. J. Cancer*, **39**, 241.

WU, R. L. & KEARNEY, R. (1980) Studies of specific tumour immunity induced with mitomycin C-treated syngeneic tumour cells (MCT). Effects of carrageenan and trypan blue on MCT-induced immunity in mice. *J. Natl Cancer Inst.*, **64**, 81.